# Influences of TP53 and the anti-aging DDR1 receptor in controlling Raf/MEK/ERK and PI3K/Akt expression and chemotherapeutic drug sensitivity in prostate cancer cell lines

**DOI:** 10.18632/aging.103377

**Published:** 2020-06-03

**Authors:** William H. Chappell, Saverio Candido, Stephen L. Abrams, Shaw M. Akula, Linda S. Steelman, Alberto M. Martelli, Stefano Ratti, Lucio Cocco, Melchiorre Cervello, Giuseppe Montalto, Ferdinando Nicoletti, Massimo Libra, James A. McCubrey

**Affiliations:** 1 Department of Microbiology and Immunology, Brody School of Medicine, East Carolina University, Greenville, NC 27834, USA; 2 Current Address: Becton, Dickinson and Company (BD), BD Diagnostics, Franklin Lakes, NJ 07417, USA; 3 Research Center for Prevention, Diagnosis and Treatment of Cancer (PreDiCT), University of Catania, Catania, Italy; 4 Department of Biomedical and Biotechnological Sciences, University of Catania, Catania, Italy; 5 Department of Biomedical and Neuromotor Sciences, Università di Bologna, Bologna, Italy; 6 Institute for Biomedical Research and Innovation, National Research Council (CNR), Palermo, Italy; 7 Department of Health Promotion, Maternal and Child Care, Internal Medicine and Medical Specialties, University of Palermo, Palermo, Italy

**Keywords:** TP53, DDR, rapamycin, RAF/MEK/ERK, collagen

## Abstract

Background: TP53 plays critical roles in sensitivity to chemotherapy, and aging. Collagen is very important in aging. The molecular structure and biochemical properties of collagen changes during aging. The discoidin domain receptor (DDR1) is regulated in part by collagen. Elucidating the links between TP53 and DDR1 in chemosensitivity and aging could improve therapies against cancer and aging.

Results: Restoration of WT-TP53 activity resulted in increased sensitivity to chemotherapeutic drugs and elevated expression of key components of the Raf/MEK/ERK, PI3K/Akt and DDR1 pathways. DDR1 could modulate the levels of Raf/MEK/ERK and PI3K/Akt pathways as well as sensitize the cells to chemotherapeutic drugs. In contrast, suppression of WT TP53 with a dominant negative (DN) TP53 gene, suppressed DDR1 protein levels and increased their chemoresistance.

Conclusion: Restoration of WT TP53 activity or increased expression of the anti-aging DDR1 collagen receptor can result in enhanced sensitivity to chemotherapeutic drugs. Our innovative studies indicate the important links between WT TP53 and DDR1 which can modulate Raf/MEK/ERK and PI3K/Akt signaling as well as chemosensitivity and aging.

Methods: We investigated the roles of wild type (WT) and mutant TP53 on drug sensitivity of prostate cancer cells and the induction of Raf/MEK/ERK, PI3K/Akt and DDR1 expression and chemosensitivity.

## INTRODUCTION

TP53 is a crucial tumor suppressor gene whose wild-type (WT) activity is lost in over 50% of human cancers. Prostate cancer is a disease of aging as most men do not develop prostate cancer until later in life (>65 years old) and is rare in men less than 40. Prostate cancer is the second leading cancer for men, behind lung cancer. Approximately 174,650 new cases of prostate cancer will be detected in the USA this year and approximately 31,620 deaths will occur (American Cancer Society). The incidence of prostate cancer as well as the prognosis depends on many factors including race, the presence of obesity and other factors such as diet [1]. Approximately 1 in 9 men will develop prostate cancer and one in forty men will die from prostate cancer. If detected early, prostate cancer can be treated by various approaches, including: androgen suppression, removal of the prostate, cryotherapy, radiation therapy, and other methods [2]. However, if prostate cancer has advanced and becomes hormone-independent, it becomes more difficult to treat effectively. In this scenario, chemotherapeutic drugs such as docetaxel and mitoxantrone and others may be used to treat the prostate cancer patient.

Various oncogenes, tumor suppressor genes and chromosomal translocations have been shown to influence the development of prostate cancer [3]. The expression of TP53 and PTEN tumor suppressors are frequently altered in prostate cancer by various mechanisms including genetic mutation and epigenetic modification. The DU145 and PC3 prostate cancer cell lines have mutations or deletions at the *TP53* gene and in some cases (e.g., PC3 cells) the *PTEN* gene. These mutations contribute to the drug-resistance and malignant properties of these cells. Previously, we determined that restoration of WT TP53 in the DU145 prostate cancer line increased the sensitivity to multiple chemotherapeutic drugs including doxorubicin, paclitaxel, cisplatin and others and increased the effectiveness of radiation treatment in inducing cellular senescence [4–6]. However, the effects of restoration of WT-TP53 on the expression of the Raf/MEK/ERK and PI3K/Akt signaling pathways are not known in cells which lack functional WT TP53.

Collagen is an important protein involved in cellular repair and aging [7]. Tumor cells are surrounded by an environment which is rich in type I collagen. Type I collagen is a major adhesive component in stroma and collagen serves to regulate proliferation and invasion. After basement-membrane degradation by tumor cells, stroma represents the first barrier against cell invasion. The molecular structure of collagen changes during aging. The structural changes of type I collagen can regulate its activities [8] The discoidin domain receptor (DDR1) is normally activated by collagen. DDR1 is involved in proliferation, cellular migration, extracellular matrix (ECM) remodeling, wound repair and other important biological processes [7]. Collagen interacts with DDR1. However, differences in the biochemical properties of adult and aged collagen influence its ability to activate DDR1. Aging results in modifications of collagen due to structural reorganization. Adult collagen will induce DDR1 which in turn induces apoptosis and inhibits cellular proliferation. In contrast, aged collagen does not induce DDR1 and hence aging and proliferation occurs which can under certain circumstance lead to cancer [8, 9]. DDR1 induces growth suppression and apoptosis by increasing the expression of the pro-apoptotic mediator BCL2-family member BIK in noninvasive luminal-like breast carcinoma cells. In contrast, membrane type-1 matrix metalloproteinase (MT1-MMP) can inhibit the effects induced by collagen/DDR1/BIK stimulation. Low levels of DDR1 have been observed during the epithelial to mesenchymal transition (EMT) process in breast cancer. Enforced overexpression of DDR1 in aggressive basal-like breast cancer cells suppressed their invasiveness in 3D culture models.

Recently, low levels of DDR1 have been associated with a poor prognosis in prostate cancer [10]. Collagen metabolism changes during prostate cancer progression [11]. While the roles of collagen, DDR1 and breast cancer invasiveness have been well investigated [8, 9, 12] the function of DDR1 in prostate cancer is not well understood.

Many commonly prescribed chemotherapeutic drugs induce reactive oxygen species (ROS) which in turn can activate signaling pathways that are often growth promoting and can lead to drug resistance. The involvement of the tumor suppressor TP53 gene product is often critically involved in the sensitivity to chemotherapeutic drugs and radiation therapy. We demonstrate for the first time that restoration of WT TP53 in prostate cancer cells which previously lacked WT TP53 activity resulted in chemosensitivity and elevated induction of the Raf/MEK/ERK, PI3K/Akt and DDR1. Likewise, in prostate cancer cell lines that normally expressed WT TP53, DDR1 was detected and its expression could be decreased by introduction of a dominant negative (DN) TP53 gene. Introduction of DDR1 into cells which lacked WT-TP53 also resulted in induction of the Raf/MEK/ERK and PI3K/Akt pathways and chemosensitivity. We observed that functional TP53 activity is associated with DDR1 expression and lost in more androgen-receptor (AR) negative prostate cancer cells. Further elucidation of the effects of TP53, PTEN, DDR1 on signaling pathways and how they alter sensitivity to therapy could resulted in enhanced treatment of patients with prostate and other cancers. Restoration of functional TP53 activity is being pursued clinically. Some of the mutant “TP53-reactivators” function via the induction of ROS [13]. Thus, the effects of restoration of functional WT TP53 activity on various signaling pathways in cells which normally lack functional TP53 remains an important area in both cancer and aging research.

**Figure 1:**
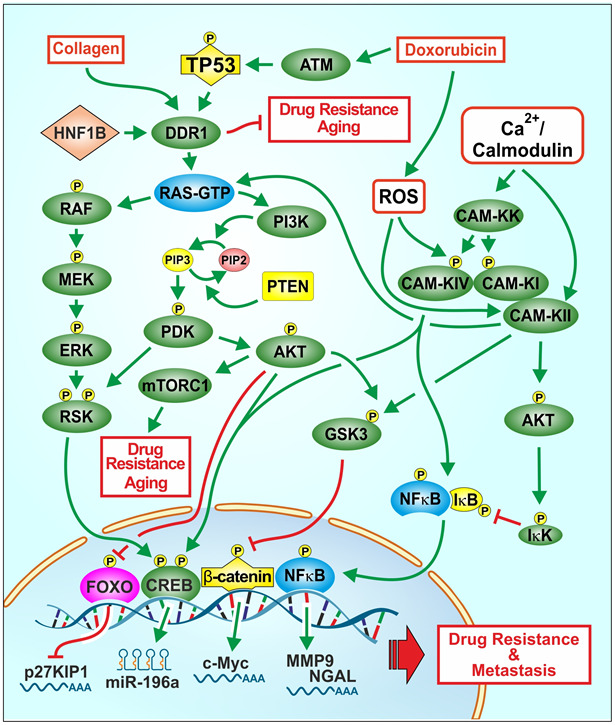
**Effects of collagen, chemotherapeutic drugs on ROS, TP53, DDR1 and downstream signaling pathways on proliferation, drug resistance and aging.** One signaling protein involved in regulating the Raf/MEK/ERK and PI3K/Akt pathways is DDR1. A ligand for DDR1 is collagen. Collagen is involved in the regulation of aging and in some cases tumorigenesis. Chemotherapeutic drugs and ROS can affect multiple signaling pathways and transcription factors such as TP53 and NF-κB which can alter the expression of proteins involved in cell growth, drug resistance and aging. ROS can induce various other signaling pathways such as Raf/MEK/ERK and PI3K/Akt which have effects on proliferation. Drug resistant cells often have altered levels of ROS. Some drug resistant cells display altered expression of drug transporters, signaling or apoptotic proteins. Treatment of cell lines with chemotherapeutic drugs frequently leads to the development of drug resistance by various mechanisms. Interactions between WT-TP53 and DDR1 and downstream Raf/MEK/ERK and PI3K/Akt pathways may have important consequences on cancer progression, drug resistance and aging.

Our studies illustrate important regulatory links between: TP53, DDR, Raf/MEK/ERK and PI3K/Akt and chemosensitivity. An overview of the effects of chemotherapeutic drugs, collagen, ROS, TP53, DDR1 on signaling pathways involved in cancer progression and drug resistance and aging is presented in Figure 1.

## RESULTS

### Characterization of prostate cancer cell lines used in this study

**Figure 2:**
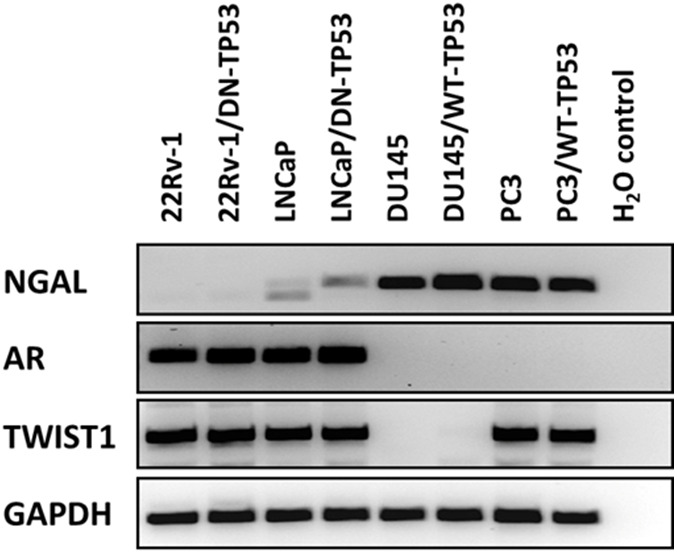
**RT-PCR analysis of NGAL, AR, TWIST1 and GAPDH mRNAs in prostate cancer cell lines containing or lacking functional TP53.** RT-PCR analysis was performed on 22Rv-1, 22Rv-1+DN-TP53, LNCaP, LNCaP + DN-TP53, DU145, DU145 + WT-TP53, PC3 and PC3 + WT-TP53 cells with oligonucleotides specific for NGAL, AR, TWIST1 and GAPDH. These genes are differentially expressed in the four prostate cancer cell lines.

Since we were examining four different prostate cancer cell lines with and without WT-TP53 activity, we determined the expression of some key genes which display altered expression in these cell lines as controls (Figure 2). Neutrophil gelatinase-associated lipocalin (NGAL) is capable of forming covalently linked complexes, both in monomeric and dimeric modes, with matrix metalloproteinase-9 (MMP-9) and is sometimes considered an accessory protein of MMP-9. NGAL is expressed at higher levels in AR- negative DU145 and PC3 cells than in AR+ 22v-1 and LNCaP cells [14]. The presence of WT-TP53 did not alter the expression of NGAL in either DU145 or PC3 cells as determined by RT-PCR. However, there were slightly higher levels of NGAL detected in LNCaP cells transfected with the dominant negative [DN] TP53. No mRNA transcripts encoding NGAL were detected in 22Rv-1 cells. The AR+ 22Rv-1 and LNCaP cells expressed mRNA transcripts encoding the AR, while the AR- DU145 and PC3 cells did not express mRNA transcripts encoding AR. The presence and absence or WT-TP53 or DN-TP53 did not alter the detection of AR mRNA transcripts. The expression of the *TWIST1* gene was examined. Interestingly, mRNA transcripts encoding TWIST1 were detected in in three of the four cell lines but not in DU145 cells. The presence and absence of WT-TP53 or DN-TP53 did not appear to change the levels of TWIST1 detected. As a loading control, the presence of GAPDH transcripts was examined and it was detected at constant levels in all cell lines in the presence or absence of WT or DN TP53.

### Effects of TP53 on DDR1, Akt and ERK in prostate cancer lines

**Figure 3:**
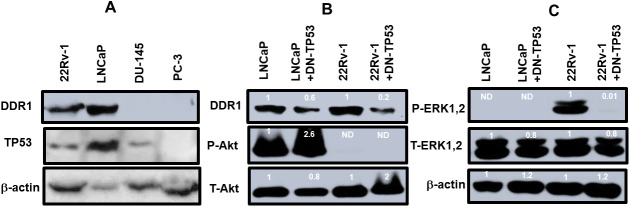
**DDR1 and TP53 expression in four prostate cancer cell lines.** Panel (**A**) The levels of DDR1, TP53 and β-actin expression were determined in 22Rv-1, LNCaP, DU145 and PC3 cells. Panel (**B**) The levels of DDR1, P-Akt and T-Akt were determined in LNCaP, LNCaP + DN-TP53, 22Rv-1 and 22Rv-1 + DN-TP53 cells. Panel (**C**) The levels of P-ERK1,2 were determined in LNCaP, LNCaP + DN-TP53, 22Rv-1 and 22Rv-1 + DN-TP53 cells. The fold values shown in white numbers and letters are presented as averages of 3 densitometric readings. Only similar cell lines are compared. ND = none detected.

The presence of DDR1 and TP53 was examined by western blot analysis in the four prostate cancer cell lines (Figure 3). DDR1 was detected in the 22Rv-1 and LNCaP cells which express functional WT TP53. In contrast, DDR1 was not detected in DU145 cells which have mutant *TP53* alleles (P223L/V274F) or PC3 cells which lack TP53. These cells have a frame shift mutation producing a stop codon on one *TP53* allele and a deletion of the other *TP53* allele (Figure 3, Panel A). The levels of TP53 were also determined in these same gels. TP53 was detected in 22Rv-1, LNCaP, DU145 but not PC3 cells. The levels of β-actin were examined as a protein loading control. β-actin was detected in all the protein samples. Interestingly, DDR1 was expressed at higher levels in cells with functional WT TP53 activity, while NGAL is expressed at lower levels in cells with functional TP53. Both DDR1 and NGAL have effects on and regulated by collagen and the ECM [7–9, 14, 15].

The effects of introduction of DN-TP53 on the levels of DDR1 in prostate cancer cell lines which normally expressed DDR1 were examined (Figure 3, Panel B). We previously demonstrated that introduction of DN-TP53 into LNCaP or 22Rv-1 cells increased their resistance to chemotherapeutic drugs [4] and ionizing radiation [5]. Introduction of DN-TP53 suppressed the levels of expression of DDR1 were 1.8- and 5-fold in LNCaP and 22Rv-1 respectively. The expression of activated Akt (S473) and total Akt were examined in these same cells. LNCaP cells have deleted *PTEN* which leads to high levels of activated Akt [16]. Upon introduction of DN-TP53 the level of activated Akt increased approximately 2.6-fold. In contrast, no activated AKT was detected in 22Rv-1 cells in the presence and absence of DN-TP53. The levels of total Akt were also examined. Introduction of DN-TP53 increased the levels of total Akt detected.

The effects of DN-TP53 on the levels of active ERK1,2 were also examined (Figure 3, Panel C). LNCaP cells do not express activated ERK1,2 because of the negative feed-back loop between high levels of activated Akt expression which suppresses activation of ERK1,2 [16]. Introduction of DN-TP53 suppressed the levels of activated ERK1,2 detected in 22Rv-1 cells. In contrast, relatively equal levels of total ERK1,2 and β-actin were detected in LNCaP and 22Rv-1 cells in the presence and absence of DN-TP53. These results serve to illustrate the effects that the presence of functional TP53 has on the expression of DDR1 and that introduction of DN-TP53 can suppress the levels of DDR1 protein detected in cells with functional WT-TP53.

### Effects of doxorubicin on induction of TP53 activity and chemosensitivity in DU145 cells containing or lacking WT TP53

**Figure 4:**
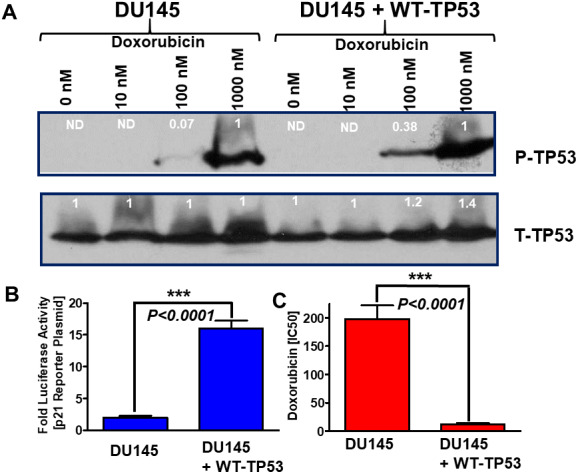
** Effects of introduction of WT-TP53 on P-TP53 protein levels, TP53-luciferase activity and doxorubicin IC50 in DU145 cells in the presence and absence of doxorubicin.** Panel (**A**) DU145 and DU145 + WT-TP53 cells were treated with different concentration of doxorubicin for 24 hours. The levels of S15-phosphorylated (active) and total TP53 were determined by western blotting. The fold values shown in white numbers and letters are presented as averages of 3 densitometric readings. ND = not detected. In the rows with S15-phosphorylated TP53, the values for DU145 and DU145 + WT-TP53 were normalized to the 1,000 nM doxorubicin treated samples as no S15-phosphorylated TP53 was detected in untreated samples. In contrast with the total (T) TP53 samples, the levels of total TP53 were normalized to the untreated samples. These experiments were repeated three times and similar results were obtained. Panel (**B**) Effects of introduction of WT TP53 on luciferase activity. The levels of luciferase activity were determined in DU145 and DU145 + WT-TP53 cells. These experiments were repeated three times and similar results were obtained. Panel (**C**). Effects of introduction of WT TP53 on sensitivity to doxorubicin were determined by MTT analysis as described [4]. These experiments were repeated six times and similar results were obtained. Statistical analysis is presented on the panel. *** = *P* < 0.0001.

The effects of different concentrations of doxorubicin on the presence of S15-phosphorylated TP53 (activated) were examined in DU145 cells which either lacked or contained WT-TP53 (Figure 4, Panel A). In cells which lacked WT TP53, S15-phosphorylated TP53 (active form) was detected after 1,000 nM doxorubicin treatment. When the DU145 cells were treated with 100 nM doxorubicin, 14.3-fold less S15-phosphorylated TP53 were detected than with 1,000 nM doxorubicin. In contrast, when DU145 + WT-TP53 cells were treated with 100 nM doxorubicin, 2.6-fold less-S15 phosphorylated TP53 were detected than with 1,000 nM doxorubicin. Thus, S15 phosphorylated TP53 was detected at higher levels in lower doxorubicin concentrations in DU145 + WT-TP53 than in DU145 cells. The levels of total TP53 were relatively constant in the cells in the presence and absence of WT-TP53.

The induction of TP53 activity was also examined with a p53 reporter luciferase construct (Figure 4, Panel B) and compared to results after transfection of the same cells with the pGL2-basic vector that contains only the luciferase gene without a promoter or enhancer (Promega, Madison, WI). This was used to determine non-specific luciferase gene background activity in all cells. The PG13-luc vector contains 13 repeats of a TP53-response element sequence obtained from the p21^Cip-1^ gene fused to the firefly luciferase gene [17]. The effects of introduction of WT-TP53 on p21-promoter based luciferase reporter construct were determined. Introduction of WT-TP53 increased p21-promoter driven luciferase assays approximately 8-fold in DU145 + WT-TP53 cells compared to DU145 cells (Panel B) demonstrating that restoration of WT-TP53 increased p21^Cip-1^ promoter activity. These results demonstrate that WT-TP53 induces classical downstream targets such as the promoter region of p21^Cip-1^.

The effects of introduction of WT-TP53 on the sensitivity of DU145 cells to doxorubicin were also determined. Introduction of WT-TP53 decreased the IC_50_ for doxorubicin approximately 16.5-fold in DU145 + WT-TP53 cells compared to DU145 cells (Panel C).

### Effects of restoration of WT-TP53 on Raf/MEK/ERK and PI3K/AKT pathways

**Figure 5:**
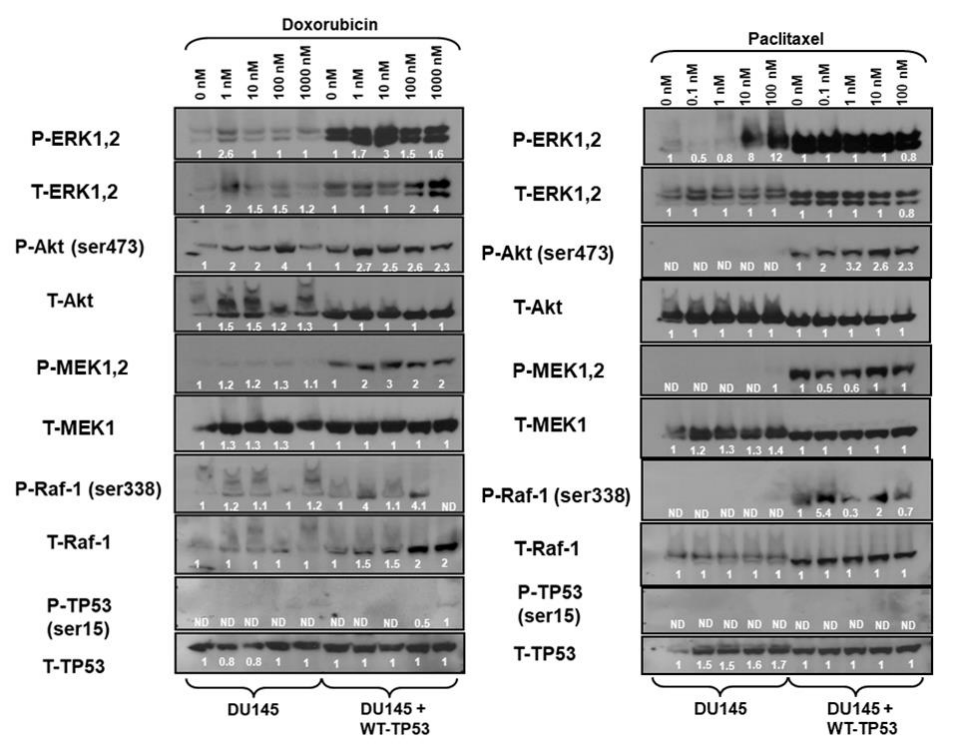
**Effects of doxorubicin and paclitaxel on the levels of the Raf/MEK/ERK, PI3K/Akt and TP53 pathways in DU145 prostate cancer cells either lacking or containing functional WT-TP53.** Western blot analysis was performed to determine the levels of key members of the Raf/MEK/ERK, PI3K/Akt and TP53 pathways in response to treatment with varying concentrations of doxorubicin or paclitaxel for 24 hours. Activation specific antibodies and antibodies measuring total levels of protein were used in these experiments. These experiments were repeated twice, and similar results were observed. The fold values shown in white numbers and letters are presented as averages of 3 densitometric readings. ND = not detected.

The effects of introduction of WT-TP53 on Raf/MEK/ERK and PI3K/Akt pathways were examined in DU145 cells. Most strikingly, introduction of WT-TP53 into TP53-deficient DU145 cells increased the levels of activated MEK, ERK and Akt detected (Figure 5). Treatment with doxorubicin resulted in activation of ERK1,2 and Akt (Figure 5, left panels). Introduction of WT-TP53 and treatment with 1,000 nM doxorubicin resulted in the induction of activated S15-phosphorylated TP53 in DU145 + WT-TP53 detected after doxorubicin treatment. The levels of the proteins were detected by densitometric scanning and normalized to the individual cell line (e.g., DU145 or DU145 + WT-TP53).

The expression of these proteins was also examined in the presence and absence of paclitaxel (Figure 5, right panels). As observed previously, increased levels of activated MEK, ERK, and Akt were detected in the DU145 cells which contained WT-TP53. Increased levels of P-Akt were observed after doxorubicin but not paclitaxel treatment of DU145 cells. Treatment of DU145 cells with 10 and 100 nM paclitaxel led to the induction of approximately 8-12-fold activated ERK expression as determined by densitometric scanning. In contrast, activation of TP53 was not observed in response to paclitaxel, while it was detected after doxorubicin treatment (Figure 5, left panel). In summary, introduction of WT-TP53 into DU145 cells resulted in higher levels of P-ERK, P-MEK expression than that detected in DU145 cells that lacked WT TP53. Paclitaxel was a potent inducer of P-ERK in DU145 cells.

### Isolation of drug resistant DU145 cells in the presence and absence of WT-TP53

**Figure 6:**
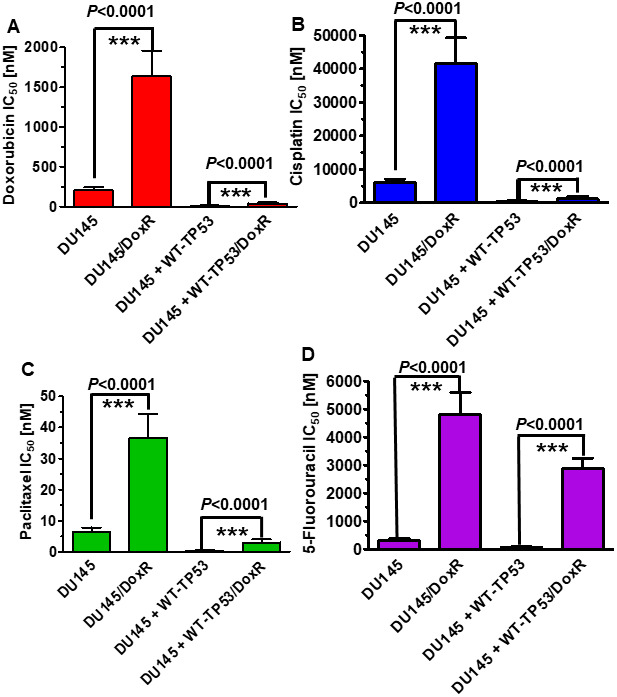
**Effects of introduction of WT-TP53 on the chemosensitivity of doxorubicin-sensitive and doxorubicin-resistant DU145 cells.** The IC_50_s of doxorubicin-sensitive and doxorubicin-resistant DU145 and DU145 + WT-TP53 cells to: Panel (**A**) Doxorubicin, Panel (**B**) Cisplatin, (**C**) Paclitaxel and (**D**) 5-Fluorouracil were determined by MTT analysis as described in Figure 4. These experiments were repeated three times and similar results were obtained. Statistical analysis is presented on the panels. *** = *P* < 0.0001.

Doxorubicin resistant DU145 cells, either containing or lacking introduced WT-TP53, were isolated by culturing the DU145 and DU145+WT-TP53 cells in the presence of 10 nM doxorubicin for two months. The sensitivities of the doxorubicin-resistant and doxorubicin-sensitive cells to doxorubicin, paclitaxel, cisplatin and 5-fluorouracil were examined and is presented in Figure 6. The doxorubicin-resistant DU145 cells were 6-11-fold more resistant to the four different chemotherapeutic drugs examined. Likewise, the doxorubicin-resistant DU145 + WT-TP53 cells were 2.5-11-fold more resistant to the four different chemotherapeutic drugs examined. The doxorubicin-resistant DU145 (DoxR) cells were more drug resistant than the DU145 + WT-TP53 (DoxR) cells which contained WT-TP53.

### Expression of Raf/MEK/ERK, PI3K/Akt and TP53 in doxorubicin resistant DU145 prostate cancer cells

**Figure 7:**
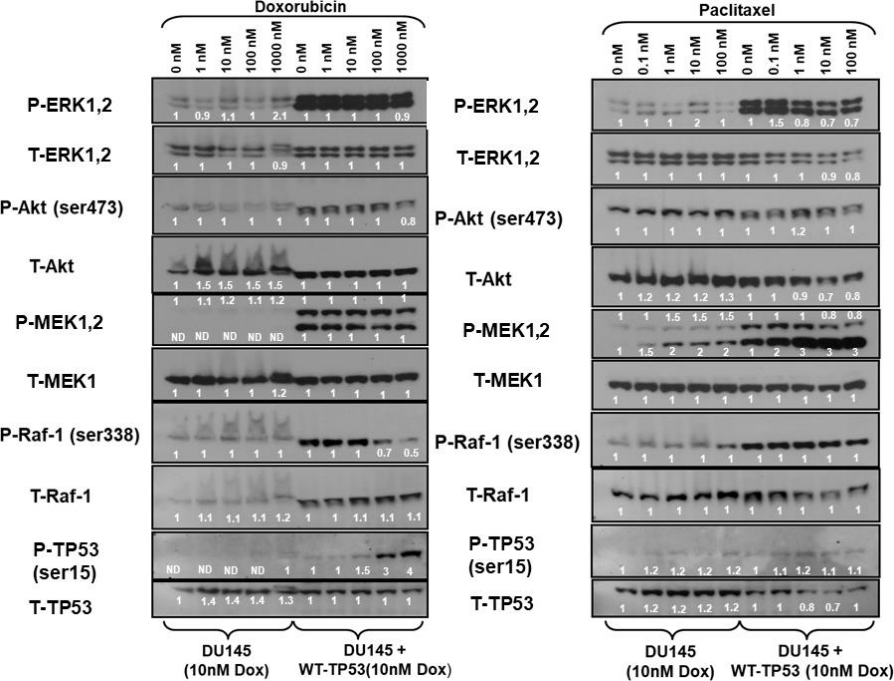
**Effects of doxorubicin and paclitaxel on the levels of the Raf/MEK/ERK, PI3K/Akt and TP53 pathways in doxorubicin-resistant DU145 prostate cancer cells either lacking or containing functional WT-TP53.** Western blot analysis was performed to determine the levels of key members of the Raf/MEK/ERK, PI3K/Akt and TP53 pathways in response to treatment with varying concentrations of doxorubicin or paclitaxel for 24 hours. Activation specific antibodies and antibodies measuring total levels of protein were used in these experiments. These experiments were repeated twice, and similar results were observed. The fold values shown in white numbers and letters are presented as averages of 3 densitometric readings. ND = not detected.

The expression of the Raf/MEK/ERK, PI3K/Akt pathways and TP53 were examined in the doxorubicin-resistant DU145 (DoxR) and DU145 + WT-TP53 (DoxR) cells either in the presence or absence of doxorubicin (Figure 7, left panel). Higher levels of activated MEK, ERK1,2, Akt, MEK and Raf were detected in cells containing WT-TP53. Induction of S15-phosphorylated TP53 was observed upon treatment of DU145 + WT-TP53 cells with 10-100 nM doxorubicin (Figure 7, left panel). In contrast, induction of S15-phosphorylated TP53 was not observed in doxorubicin-resistant DU145 cells which lacked WT TP53.

The expression of the Raf/MEK/ERK, PI3K/Akt pathways and TP53 were examined in the doxorubicin-resistant DU145 (DoxR) and DU145 + WT-TP53 (DoxR) cells either in the presence or absence of paclitaxel (Figure 7, right panel). Higher levels of activated ERK, MEK and Raf were detected in doxorubicin-resistant DU145 cells containing WT-TP53 than in doxorubicin-resistant DU145 cells lacking WT-TP53. The phospho-specific MEK1/2 antibody (Ab) often detects two bands (MEK1 and MEK2). In these blots where both bands detected with the phospho-specific MEK1/2 Ab, the changes in intensity of both bands were determined. The pronounced induction of S15-phosphorylated TP53 detected after doxorubicin treatment was not observed as was detected after paclitaxel treatment of doxorubicin-resistant DU145 + WT-TP53 cells (Figure 7). In summary of the western blot protein data presented, introduction of WT-TP53 into DU145 cells increased the expression of active MEK, ERK. Doxorubicin-resistant DU145 + WT-TP53 cells displayed increased levels of S15-phosphorylated TP53 when the cells were treated doxorubicin but not paclitaxel.

### Effects of restoration of WT-TP53 activity on DDR1 expression in DU145 prostate cancer cells

**Figure 8:**
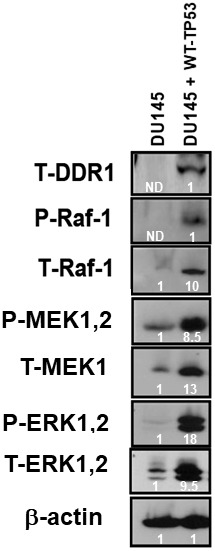
**Effects of restoration of WT-TP53 on DDR1 and Raf/MEK/ERK protein levels in DU145 prostate cancer cells either lacking or containing functional WT-TP53.** Western blot analysis was performed to determine the levels DDR1 and key members of the Raf/MEK/ERK pathway in response to restoration of WT-TP53. Levels of actin were determined as a protein loading control These experiments were repeated twice, and similar results were observed. The fold values shown in white numbers and letters are presented as averages of 3 densitometric readings. ND = not detected.

In Figure 3, we demonstrated links between TP53 and DDR1 expression in four prostate cancer cell lines. Links between the TP53, DDR1 and Raf/MEK/ERK pathways have been observed in various other cancer cells including osteosarcoma and breast cancer cells [18]. To further ascertain whether there were associations between TP53, DDR1, and the RAS/Raf/MEK/ERK cascade in DU145 prostate cancer cells either lacking or containing WT-TP53, the expression of DDR1 was monitored in DU145 and DU145 + WT-TP53 cells (Figure 8). The levels of DDR1, Raf1, MEK, ERK were all higher in the DU145 + WT-TP53 than in DU145 cells lacking WT-TP53. Restoration of WT-TP53 resulted in the expression of DDR1, and as demonstrated previously, the Raf/MEK/ERK cascade.

### Effects of introduction of a retrovirus encoding DDR1b on Raf/MEK/ERK and PI3K/Akt protein levels and activation

**Figure 9:**
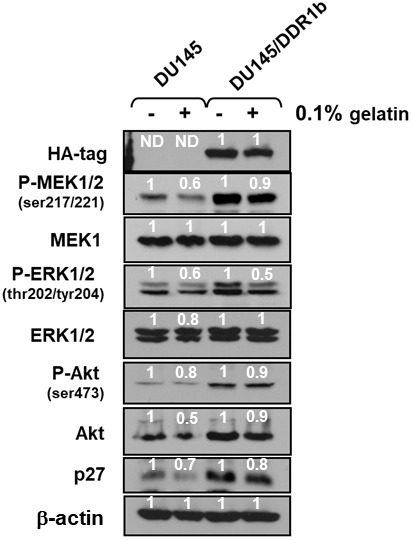
**Effects of introduction of DDR1b on Raf/MEK/ERK and Akt protein levels in DU145 prostate cancer cells.** Western blot analysis was performed to determine the levels DDR1 and key members of the Raf/MEK/ERK pathway and Akt and p27^Kip-1^ in response to introduction of DDR1b. Cells were cultured in the presence and absence of 0.1 gelatin. The levels of DDR1b were determined upon analysis with an HA-tag antibody as the DDR1b encoding retrovirus has a HA-tag. Levels of βactin were determined as a protein loading control These experiments were repeated twice, and similar results were observed. The fold values shown in white numbers and letters are presented as averages of 3 densitometric readings. ND = not detected.

To determine further whether there were links between DDR1 and the Raf/MEK/ERK and PI3K/Akt pathways, the effects of introduction of a retrovirus encoding DDR1b [19] on the expression of these pathways were determined. Introduction of DDR1b into DU145 cells resulted in increased expression of activated MEK1, ERK1 and Akt as well as cyclin-dependent kinase inhibitor 1B (p27^Kip1^) (Figure 9). Akt has been shown to regulate the levels of p27^KIP1^ [20]. In these cells, DDR1b was detected at high levels with the HA-tag antibody. Gelatin is the cooked/processed form of collagen [21] which can activate DDR1. Treatment with 0.1% gelatin resulted in decreased levels of active ERK1,2, MEK1,2, Akt and p27^Kip1^. The reduction of expression of these molecules was observed both in DU145 and DU145 + WT-TP53 cells. Thus, in this model system, introduction of DDR1b increased the expression of these genes and treatment with gelatin decreased the expression of these genes which are often associated with proliferation. There are likely other receptors which are regulated by gelatin/collagen and DDR1b is a member of a multigene family [22].

**Figure 10:**
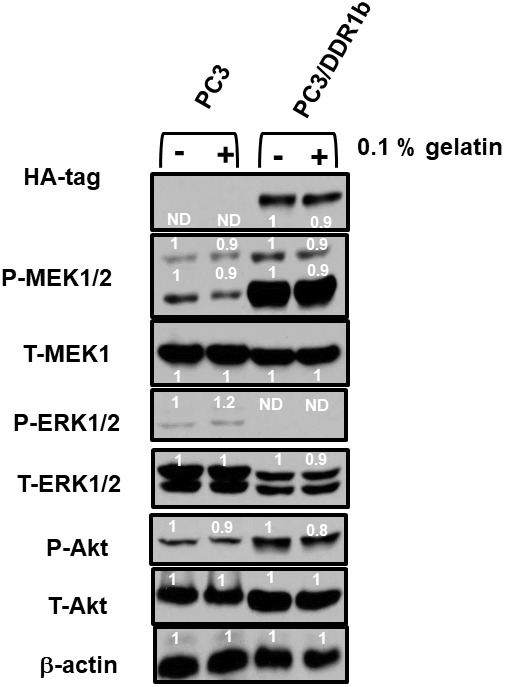
**Effects of introduction of DDR1b on Raf/MEK/ERK and Akt protein levels in PC3 Prostate cancer cells.** Western blot analysis was performed to determine the levels DDR1 and key members of the RAF/MEK/ERK pathway and Akt ^in^ response to introduction of DDR1b. Cells were cultured in the presence and absence of 0.1 gelatin which in some circumstances activates DDR1. The levels of DDR1b were determined upon analysis with an HA-tag antibody as the DDR1b encoding retrovirus has a HA-tag. Levels of β-actin were determined as a protein loading control These experiments were repeated twice, and similar results were observed. The fold values shown in white numbers and letters are presented as averages of 3 densitometric readings. ND = not detected.

The effects of DDR1b overexpression were also examined on the PC3 prostate cancer cell line (Figure 10). Introduction of DDR1b resulted in higher levels of active MEK1,2 and AKT expression than that observed in PC3 cells. Interestingly, introduction of DDR1 reduced the levels of active and total ERK expression detected. Treatment of the PC3 and PC3/DDR1b cells with gelatin reduced the levels of active MEK and Akt expression.

### Effects of introduction of a retrovirus encoding DDR1b on chemosensitivity

**Figure 11:**
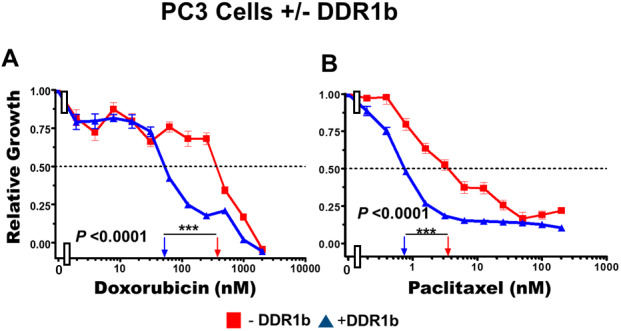
**Effects of introduction of DDR1b on chemosensitivity of PC3 cells.** The effects of introduction of DDR1b on the chemosensitivity of PC3 cells to: doxorubicin (Panel A) and paclitaxel (Panel B) was determined by MTT analysis as described in Figure 4. Statistical analysis is presented on the figure. *** = *P* < 0.0001.

Introduction of DDR1b increased the sensitivity of these cells to doxorubicin approximately 8-fold (Figure 11, Panel A) and paclitaxel approximately 5-fold (Figure 11, Panel B). Thus, expression of DDR1b can result in sensitization of PC3 prostate cancer cells to doxorubicin and paclitaxel.

## DISCUSSION

Previously, we determined that introduction of WT-TP53 into DU145 prostate cancer cells which lacked functional WT TP53 resulted in increased sensitivity to chemotherapeutic drugs and ionizing radiation [4, 5]. Introduction of WT-TP53 into DU145 cells resulted in an 8-fold elevation in p53 luciferase activity and increased the sensitivity to doxorubicin approximately 20-fold. On the other hand, introduction of a DN-TP53 gene into LNCaP and 22RV-1 prostate cancer cells, which contain WT TP53, resulted in decreased sensitivity to chemotherapeutic drugs and ionizing radiation [4, 5]. The IC_50_s for doxorubicin was increased approximately 4-fold in LNCaP + DN-TP53 cells. Introduction of DN-TP53 into LNCaP cells resulted in approximately 34-fold decrease in TP53 luciferase activity [4]. In the studies presented in this manuscript, we determined that introduction of DN-TP53 into LNCaP and 22Rv-1 cells resulted in reduced levels of DDR1.

The presence of WT TP53 activity is an important determinate in chemosensitivity and radio-sensitivity and DDR1 levels. Intriguingly, we observed in our studies that restoration of WT-TP53 activity increased the levels of Raf/MEK/ERK, Akt and DDR1. Augmented expression of these proteins was associated with increased chemotherapeutic drug sensitivity.

TP53 is a crucial metabolic switch involved cellular functions and survival [23–26]. Altered metabolism is an important component in cancer progression. TP53 is a major regulator of metabolic processes and is linked with essential mitochondrial events associated with cell survival. TP53 regulates the expression of many molecules which function at the mitochondrial to regulate apoptosis. TP53 downregulates many essential components of the glycolytic pathway, including glucose entry into cells [23–26]. A consequence of restoration of WT TP53 in DU145 cells could be decreased glucose uptake, inhibition of proliferation and drug sensitivity. TP53 inhibits glycolysis by suppressing hexokinase, GLUT1 and GLUT4 activity and stimulates TP53-induced glycolysis and apoptosis regulator (TIGAR) expression which is a regulator of glucose breakdown. TIGAR functions by blocking glycolysis. TIGAR also protects cells from DNA damaging ROS and DNA damage-induced apoptosis. By promoting glutamine metabolism, TP53 stimulates increased oxidative phosphorylation (OXPHOS). Dysregulated cellular metabolism is often associated with drug resistance [25]. In contrast, restoration of TP53 could result in increased OXPHOS. In breast cancer cells, induction of TP53 blocks cell cycle progression, and increases oxidative respiration and mitochondria biogenesis [27, 28]. Further studies with the doxorubicin sensitive and resistant DU145 and DU145 + WT-TP53 cells could provide more information regarding the roles of TP53 and DDR1 in regulation of metabolic processes associated with cell survival and drug resistance.

Our studies point to potential the utility of restoring WT TP53 activity in human cancers. Mutant TP53 reactivators have been developed and some are being evaluated in clinical trials [29]. The activity of certain mutant TP53 activators such as APR-246 (Prima-1Met) in some cells is dependent upon ROS levels [30].

ROS can also modulate the activity of multiple signaling pathways which are involved in growth, apoptosis and drug resistance (Figure 1) [31–33]. ROS can induce multiple signaling pathways including the CAMK cascade [34–37].

The calcium/calmodulin-dependent protein kinase (CAMK) cascade has been demonstrated to induce multiple signaling and anti-apoptotic pathways including Ras/Raf/MEK/ERK, PI3K/Akt/GSK-3 and NF-κB [34–37]. CAMKs have other multifunctional targets such as glycogen synthase kinase 3 (GSK-3) which plays important roles in multiple diseases and metabolic processes [38, 39].

DDR1 has been shown to have effects on various molecules involved in prostate cancer metastasis such as MMPs [40]. We chose to examine the effects of DDR1b expression in PC3 cells as they lack WT TP53, AR and PTEN activity and have high levels of activated Akt but very low levels of ERK1,2 pathway activation due to elevated Akt activity in these cells [16]. Previously we determined that PC3 cells expressed high levels of NGAL and MMP9 and that suppression of NGAL inhibited the colony formation of the cells in soft agar [14]. In this current study, we observed that introduction of DDR1 into PC3 cells increased their chemosensitivity.

DDR1 expression is one of twelve genes identified in a study to be associated with HNF1 homeobox B (HNF1b) mediated prostate cancer risk [41]. HNF1b is a transcription factor which is associated with the development of many cancers. One target gene of both *HNF1b* and *TP53* is *DDR1* [41]. Combined *HNF1B* and *TP53* loss has been proposed to enhance cellular survival and result in an aggressive phenotype in certain cancers [42]. In contrast, restoration of WT TP53 increased the chemosensitivity of various cancer including prostate and pancreatic cancers [5, 6, 43]. DDR1 increased the chemosensitivity of prostate cancer cells. DDR1 may have some anti-aging functions and may be activated by DDR1, TP53, Ras/Raf/MEK/ERK, and PI3K/Akt and how they are involved in regulation of drug- sensitivity. Restoration of WT TP53 activity resulted in increased expression of these pathways as well as increased drug-sensitivity. Suppression of WT TP53 with a DN-TP53 gene in cells that normally express WT TP53 resulted in decreased DDR1 levels and chemoresistance.

The effects of DDR1 may vary depending on the cell type. In studies with breast cancer cells, DDR1 was associated with induction of apoptosis [8, 9, 12]. While suppression of DDR1 has also been observed to reduce the invasive properties of other cancer cell lines such as melanoma, colon and hepatoma cells [44]. Interestingly, DDR1b and DDR2, promoted tumor growth in the presence of collagen while DDR1b suppressed the lung metastasis of HT1080 fibrosarcoma cells in a xenograft model [45]. Elucidating the interactions between TP53 and DDR1 and drug sensitivity could further our understanding of how the invasive properties of various cancer types is influenced by the presence of different types/classes of adult vs old collagen present in the extracellular matrix.

## MATERIALS AND METHODS

### Cell culture

LNCaP, 22Rv-1, DU145 and PC3 cells were purchased from the ATCC (Rockville, MD, USA) and maintained in RPMI + 10% fetal bovine serum (FBS) (Atlanta Biologicals, Atlanta, GA, USA) and antibiotics (Atlanta Biologicals) as described previously [5, 6, 14]. Gelatin was purchased from Invitrogen (Carlsbad, CA)

### Retroviral infection

The DDR1b retrovirus was generously provided by Dr. Elaine W. Raines, University of Washington [19]. The gene encoding the DDR1b is HA tagged, which makes identification of the introduced DDR1 protein readily detectable. Cell were infected with the DDR1b as previously described [4, 5, 46]. Stably-infected cells were isolated in RPMI + 10% FBS containing two micrograms/ml puromycin (Sigma-Aldrich, Saint Louis, MO) as the DDR1b cDNA was cloned in the pBM-IRESPURO vector [19]. The WT-TP53 and DN-TP53 retrovirus vectors were generously provided by Dr. Moshe Oren, Weizmann Institute of Science, Israel [47, 48].

### Western blotting

Western blotting was performed as described [5, 6, 46]. Most antibodies were purchased from Cell Signaling (Beverly, MA, USA) with the exception of the anti-DDR1 antibody which was purchased from Santa Cruz Biotechnology (Santa Cruz, CA, USA) and the anti HA antibody which was purchased from Zymed laboratories (South San Francisco, CA, USA).

### Luciferase assays

Luciferase reporter plasmids were used to measure both endogenous and exogenous TP53 promoter activity DU145 and DU145 + WT-TP53 cells. The pGL2-basic vector contains only the luciferase gene without a promoter or enhancer and was used to determine non-specific luciferase gene background activity in both types of cells (Promega, Madison, WI). The PG13-luc vector contain thirteen repeats of the TP53 response element sequence present in the *CDKN1* (p21^CIP-1^) gene fused to the firefly luciferase gene [17]. As a control for transfection efficiency, the CMV-β-galactosidase expression plasmid was used. It contains a β-galactosidase gene regulated by a cytomegalovirus promoter [49].

7 x 10^5^ total cells were plated per well of a six-well plate in RPMI + 10% FBS and allowed to attach overnight under normal culture conditions. The next day, cells were cotransfected with three μg of the appropriate luciferase vector along with one μg of the CMVβ-galactosidase vector using Lipofectin™ (Invitrogen™, Carlsbad, CA). Cells were harvested forty-eight hours later and analyzed for both luciferase and β-galactosidase activity. Enzyme activity was determined using the luciferase assay system and β-galactosidase kits (Promega, Madison, WI). Luciferase readings were obtained from a TD-20/20 luminometer (Turner Designs, Sunnyvale, CA) and normalized to β-galactosidase readings obtained from the same sample. Normalized values were then calculated and graphed as fold luciferase activity over normalized luciferase values from cells transfected with the pGL2-basic vector.

### RT-PCR

RNA isolation was performed as described [14]. Briefly 200 μL of TRIzol reagent (Invitrogen) was mixed with the cell pellet and allowed to incubate for five minutes at room temperature. Then 40μL of chloroform (Fisher Scientific, Fair Lawn, NJ) was added and samples stored in a -80C freezer. 500 ng of RNA was used to produce complementary DNA (cDNA) with the iScript™ cDNA synthesis kit (Bio-Rad, Hercules, CA). Two μL of the resulting cDNA was used in PCR reactions with Illustra™ puReTaq ready-to-go PCR beads (GE Healthcare, Piscataway, NJ). Primers for specific genes included NGAL- F–TCACCTCCGTCCTGTTTAGG, R – CGAAGTCAGCTCCTTGGTC, AR-F-CTCTCTCAAGAGTTTGGATGGCT R – CACTTGCACAGAGATCATCTCTGC, GAPDH-F-ATGGCCTTCCGTGTCCCCACTG R – TGAGTGTGGCAGGGACTCCCCA, TWIST1-F-TCCTCTACCAGGTCCTCCA; TWIST1-R-GAACAATGACATCTAGGTCTC. PCR products were electrophoresed on 2% agarose gels and visualized after staining the gels with ethidium bromide.

### MTT and statistical analysis

MTT analysis was performed as described [5, 6] and statistically analyzed with Graph Pad Prism as described [50].

## References

[1] Amling CL, Riffenburgh RH, Sun L, Moul JW, Lance RS, Kusuda L, Sexton WJ, Soderdahl DW, Donahue TF, Foley JP, Chung AK, McLeod DG. Pathologic variables and recurrence rates as related to obesity and race in men with prostate cancer undergoing radical prostatectomy. J Clin Oncol. 2004; 22:439–45. 10.1200/JCO.2004.03.132 14691120

[2] Chappell WH, Abrams SL, Lertpiriyapong K, Fitzgerald TL, Martelli AM, Cocco L, Rakus D, Gizak A, Terrian D, Steelman LS, McCubrey JA. Novel roles of androgen receptor, epidermal growth factor receptor, TP53, regulatory RNAs, NF-kappa-B, chromosomal translocations, neutrophil associated gelatinase, and matrix metalloproteinase-9 in prostate cancer and prostate cancer stem cells. Adv Biol Regul. 2016; 60:64–87. 10.1016/j.jbior.2015.10.001 26525204

[3] McCubrey JA, Abrams SL, Umezawa K, Cocco L, Martelli AM, Franklin RA, Chappell WH, Steelman LS. Novel approaches to target cancer initiating cells-eliminating the root of the cancer. Adv Biol Regul. 2012; 52:249–64. 10.1016/j.advenzreg.2011.09.011 21930143

[4] Chappell WH, Lehmann BD, Terrian DM, Abrams SL, Steelman LS, McCubrey JA. P53 expression controls prostate cancer sensitivity to chemotherapy and the MDM2 inhibitor nutlin-3. Cell Cycle. 2012; 11:4579–88. 10.4161/cc.22852 23187804 PMC3562303

[5] Lehmann BD, McCubrey JA, Jefferson HS, Paine MS, Chappell WH, Terrian DM. A dominant role for p53-dependent cellular senescence in radiosensitization of human prostate cancer cells. Cell Cycle. 2007; 6:595–605. 10.4161/cc.6.5.3901 17351335

[6] Lehmann BD, McCubrey JA, Terrian DM. Radiosensitization of prostate cancer by priming the wild-type p53-dependent cellular senescence pathway. Cancer Biol Ther. 2007; 6:1165–70. 10.4161/cbt.6.8.4544 18059157 PMC2889025

[7] Huang Y, Arora P, McCulloch CA, Vogel WF. The collagen receptor DDR1 regulates cell spreading and motility by associating with myosin IIA. J Cell Sci. 2009; 122:1637–46. 10.1242/jcs.046219 19401332

[8] Saby C, Collin G, Sinane M, Buache E, Van Gulick L, Saltel F, Maquoi E, Morjani H. DDR1 and MT1-MMP expression levels are determinant for triggering BIK-mediated apoptosis by 3D type I collagen matrix in invasive basal-like breast carcinoma cells. Front Pharmacol. 2019; 10:462. 10.3389/fphar.2019.00462 31130862 PMC6509437

[9] Saby C, Rammal H, Magnien K, Buache E, Brassart-Pasco S, Van-Gulick L, Jeannesson P, Maquoi E, Morjani H. Age-related modifications of type I collagen impair DDR1-induced apoptosis in non-invasive breast carcinoma cells. Cell Adh Migr. 2018; 12:335–47. 10.1080/19336918.2018.1472182 29733741 PMC6363044

[10] Bonfil RD, Sohail A, Vranić S, Oliveira DS, Shi D, Chen W, Jang H, Saliganan AD, Wasinski BD, Kim HRC, Fridman RA. Discoidin domain receptor 1 (DDR1): A potential suppressor of prostate cancer progression. Cancer Research. 2018; 78:abstract 1070 10.1158/1538-7445.AM2018-1070

[11] Burns-Cox N, Avery NC, Gingell JC, Bailey AJ. Changes in collagen metabolism in prostate cancer: a host response that may alter progression. J Urol. 2001; 166:1698–701. 10.1016/s0022-5347(05)65656-x 11586205

[12] Jing H, Song J, Zheng J. Discoidin domain receptor 1: new star in cancer-targeted therapy and its complex role in breast carcinoma. Oncol Lett. 2018; 15:3403–08. 10.3892/ol.2018.7795 29467865 PMC5795932

[13] Yin ZX, Hang W, Liu G, Wang YS, Shen XF, Sun QH, Li DD, Jian YP, Zhang YH, Quan CS, Zeng Q, Li YL, Zhao RX, et al. PARP-1 inhibitors sensitize HNSCC cells to APR-246 by inactivation of thioredoxin reductase 1 (TrxR1) and promotion of ROS accumulation. Oncotarget. 2017; 9:1885–97. 10.18632/oncotarget.21277 29416738 PMC5788606

[14] Chappell WH, Candido S, Abrams SL, Russo S, Ove R, Martelli AM, Cocco L, Ramazzotti G, Cervello M, Montalto G, Steelman LS, Leng X, Arlinghaus RB, et al. Roles of p53, NF-κB and the androgen receptor in controlling NGAL expression in prostate cancer cell lines. Adv Biol Regul. 2018; 69:43–62. 10.1016/j.jbior.2018.05.002 29861174

[15] Hu L, Hittelman W, Lu T, Ji P, Arlinghaus R, Shmulevich I, Hamilton SR, Zhang W. NGAL decreases e-cadherin-mediated cell-cell adhesion and increases cell motility and invasion through Rac1 in colon carcinoma cells. Lab Invest. 2009; 89:531–48. 10.1038/labinvest.2009.17 19308044 PMC7770608

[16] Lee JT, Steelman LS, Chappell WH, McCubrey JA. Akt inactivates ERK causing decreased response to chemotherapeutic drugs in advanced CaP cells. Cell Cycle. 2008; 7:631–36. 10.4161/cc.7.5.5416 18256541

[17] el-Deiry WS, Tokino T, Velculescu VE, Levy DB, Parsons R, Trent JM, Lin D, Mercer WE, Kinzler KW, Vogelstein B. WAF1, a potential mediator of p53 tumor suppression. Cell. 1993; 75:817–25. 10.1016/0092-8674(93)90500-p 8242752

[18] Ongusaha PP, Kim JI, Fang L, Wong TW, Yancopoulos GD, Aaronson SA, Lee SW. P53 induction and activation of DDR1 kinase counteract p53-mediated apoptosis and influence p53 regulation through a positive feedback loop. EMBO J. 2003; 22:1289–301. 10.1093/emboj/cdg129 12628922 PMC151063

[19] Ferri N, Carragher NO, Raines EW. Role of discoidin domain receptors 1 and 2 in human smooth muscle cell-mediated collagen remodeling: potential implications in atherosclerosis and lymphangioleiomyomatosis. Am J Pathol. 2004; 164:1575–85. 10.1016/S0002-9440(10)63716-9 15111304 PMC1615659

[20] van Duijn PW, Trapman J. PI3K/akt signaling regulates p27(kip1) expression via Skp2 in PC3 and DU145 prostate cancer cells, but is not a major factor in p27(kip1) regulation in LNCaP and PC346 cells. Prostate. 2006; 66:749–60. 10.1002/pros.20398 16425184

[21] Allan JA, Docherty AJ, Barker PJ, Huskisson NS, Reynolds JJ, Murphy G. Binding of gelatinases a and B to type-I collagen and other matrix components. Biochem J. 1995; 309:299–306. 10.1042/bj3090299 7619071 PMC1135833

[22] Carafoli F, Hohenester E. Collagen recognition and transmembrane signalling by discoidin domain receptors. Biochim Biophys Acta. 2013; 1834:2187–94. 10.1016/j.bbapap.2012.10.014 23128141 PMC4332414

[23] Matoba S, Kang JG, Patino WD, Wragg A, Boehm M, Gavrilova O, Hurley PJ, Bunz F, Hwang PM. P53 regulates mitochondrial respiration. Science. 2006; 312:1650–53. 10.1126/science.1126863 16728594

[24] Vousden KH, Ryan KM. P53 and metabolism. Nat Rev Cancer. 2009; 9:691–700. 10.1038/nrc2715 19759539

[25] Bosc C, Selak MA, Sarry JE. Resistance is futile: targeting mitochondrial energetics and metabolism to overcome drug resistance in cancer treatment. Cell Metab. 2017; 26:705–07. 10.1016/j.cmet.2017.10.013 29117545

[26] Moulder DE, Hatoum D, Tay E, Lin Y, McGowan EM. The roles of p53 in mitochondrial dynamics and cancer metabolism: the pendulum between survival and death in breast cancer? Cancers (Basel). 2018; 10:189. 10.3390/cancers10060189 29890631 PMC6024909

[27] McGowan EM, Alling N, Jackson EA, Yagoub D, Haass NK, Allen JD, Martinello-Wilks R. Evaluation of cell cycle arrest in estrogen responsive MCF-7 breast cancer cells: pitfalls of the MTS assay. PLoS One. 2011; 6:e20623. 10.1371/journal.pone.0020623 21673993 PMC3108819

[28] McGowan EM, Tran N, Alling N, Yagoub D, Sedger LM, Martiniello-Wilks R. p14ARF post-transcriptional regulation of nuclear cyclin D1 in MCF-7 breast cancer cells: discrimination between a good and bad prognosis? PLoS One. 2012; 7:e42246. 10.1371/journal.pone.0042246 22860097 PMC3408480

[29] Deneberg S, Cherif H, Lazarevic V, Andersson PO, von Euler M, Juliusson G, Lehmann S. An open-label phase I dose-finding study of APR-246 in hematological Malignancies. Blood Cancer J. 2016; 6:e447. 10.1038/bcj.2016.60 27421096 PMC5141349

[30] Bykov VJ, Zhang Q, Zhang M, Ceder S, Abrahmsen L, Wiman KG. Targeting of mutant p53 and the cellular redox balance by APR-246 as a strategy for efficient cancer therapy. Front Oncol. 2016; 6:21. 10.3389/fonc.2016.00021 26870698 PMC4737887

[31] Yokoyama C, Sueyoshi Y, Ema M, Mori Y, Takaishi K, Hisatomi H. Induction of oxidative stress by anticancer drugs in the presence and absence of cells. Oncol Lett. 2017; 14:6066–70. 10.3892/ol.2017.6931 29113247 PMC5661396

[32] Kumari S, Badana AK, G MM, G S, Malla R. Reactive oxygen species: a key constituent in cancer survival. Biomark Insights. 2018; 13:1177271918755391. 10.1177/1177271918755391 29449774 PMC5808965

[33] Deben C, Deschoolmeester V, De Waele J, Jacobs J, Van den Bossche J, Wouters A, Peeters M, Rolfo C, Smits E, Lardon F, Pauwels P. Hypoxia-induced cisplatin resistance in non-small cell lung cancer cells is mediated by HIF-1α and mutant p53 and can be overcome by induction of oxidative stress. Cancers (Basel). 2018; 10:126. 10.3390/cancers10040126 29690507 PMC5923381

[34] Franklin RA, Rodriguez-Mora OG, Lahair MM, McCubrey JA. Activation of the calcium/calmodulin-dependent protein kinases as a consequence of oxidative stress. Antioxid Redox Signal. 2006; 8:1807–17. 10.1089/ars.2006.8.1807 16987033

[35] Lahair MM, Howe CJ, Rodriguez-Mora O, McCubrey JA, Franklin RA. Molecular pathways leading to oxidative stress-induced phosphorylation of akt. Antioxid Redox Signal. 2006; 8:1749–56. 10.1089/ars.2006.8.1749 16987028

[36] McCubrey JA, Lahair MM, Franklin RA. Reactive oxygen species-induced activation of the MAP kinase signaling pathways. Antioxid Redox Signal. 2006; 8:1775–89. 10.1089/ars.2006.8.1775 16987031

[37] Rodriguez-Mora O, LaHair MM, Howe CJ, McCubrey JA, Franklin RA. Calcium/calmodulin-dependent protein kinases as potential targets in cancer therapy. Expert Opin Ther Targets. 2005; 9:791–808. 10.1517/14728222.9.4.791 16083343

[38] Song B, Lai B, Zheng Z, Zhang Y, Luo J, Wang C, Chen Y, Woodgett JR, Li M. Inhibitory phosphorylation of GSK-3 by CaMKII couples depolarization to neuronal survival. J Biol Chem. 2010; 285:41122–34. 10.1074/jbc.M110.130351 20841359 PMC3003410

[39] Duda P, Akula SM, Abrams SL, Steelman LS, Martelli AM, Cocco L, Ratti S, Candido S, Libra M, Montalto G, Cervello M, Gizak A, Rakus D, McCubrey JA. Targeting GSK3 and associated signaling pathways involved in cancer. Cells. 2020; 9:E1110. 10.3390/cells9051110 32365809 PMC7290852

[40] Reel B, Korkmaz CG, Arun MZ, Yildirim G, Ogut D, Kaymak A, Micili SC, Ergur BU. The regulation of matrix metalloproteinase expression and the role of discoidin domain receptor 1/2 signalling in zoledronate-treated PC3 cells. J Cancer. 2015; 6:1020–29. 10.7150/jca.12733 26366216 PMC4565852

[41] Hu YL, Zhong D, Pang F, Ning QY, Zhang YY, Li G, Wu JZ, Mo ZN. HNF1b is involved in prostate cancer risk via modulating androgenic hormone effects and coordination with other genes. Genet Mol Res. 2013; 12:1327–35. 10.4238/2013.April.25.4 23661456

[42] Sun M, Tong P, Kong W, Dong B, Huang Y, Park IY, Zhou L, Liu XD, Ding Z, Zhang X, Bai S, German P, Powell R, et al. HNF1B loss exacerbates the development of chromophobe renal cell carcinomas. Cancer Res. 2017; 77:5313–26. 10.1158/0008-5472.CAN-17-0986 28807937 PMC5626626

[43] Abrams SL, Lertpiriyapong K, Yang LV, Martelli AM, Cocco L, Ratti S, Falasca M, Murata RM, Rosalen PL, Lombardi P, Libra M, Candido S, Montalto G, et al. Introduction of WT-TP53 into pancreatic cancer cells alters sensitivity to chemotherapeutic drugs, targeted therapeutics and nutraceuticals. Adv Biol Regul. 2018; 69:16–34. 10.1016/j.jbior.2018.06.002 29980405

[44] Romayor I, Badiola I, Olaso E. Inhibition of DDR1 reduces invasive features of human A375 melanoma, HT29 colon carcinoma and SK-HEP hepatoma cells. Cell Adh Migr. 2020; 14:69–81. 10.1080/19336918.2020.1733892 32090682 PMC7153652

[45] Wasinski B, Sohail A, Bonfil RD, Kim S, Saliganan A, Polin L, Bouhamdan M, Kim HC, Prunotto M, Fridman R. Discoidin domain receptors, DDR1b and DDR2, promote tumour growth within collagen but DDR1b suppresses experimental lung metastasis in HT1080 xenografts. Sci Rep. 2020; 10:2309. 10.1038/s41598-020-59028-w 32047176 PMC7012844

[46] Chappell WH, Abrams SL, Stadelman KM, LaHair MM, Franklin RA, Cocco L, Evangelisti C, Chiarini F, Martelli AM, Steelman LS, McCubrey JA. Increased NGAL (Lnc2) expression after chemotherapeutic drug treatment. Adv Biol Regul. 2013; 53:146–55. 10.1016/j.jbior.2012.09.004 23073564

[47] Eliyahu D, Michalovitz D, Eliyahu S, Pinhasi-Kimhi O, Oren M. Wild-type p53 can inhibit oncogene-mediated focus formation. Proc Natl Acad Sci USA. 1989; 86:8763–67. 10.1073/pnas.86.22.8763 2530586 PMC298370

[48] Shaulian E, Zauberman A, Ginsberg D, Oren M. Identification of a minimal transforming domain of p53: negative dominance through abrogation of sequence-specific DNA binding. Mol Cell Biol. 1992; 12:5581–92. 10.1128/mcb.12.12.5581 1448088 PMC360497

[49] Madrid LV, Mayo MW, Reuther JY, Baldwin AS Jr. Akt stimulates the transactivation potential of the RelA/p65 subunit of NF-kappa B through utilization of the ikappa B kinase and activation of the mitogen-activated protein kinase p38. J Biol Chem. 2001; 276:18934–40. 10.1074/jbc.M101103200 11259436

[50] Akula SM, Candido S, Libra M, Abrams SL, Steelman LS, Lertpiriyapong K, Ramazzotti G, Ratti S, Follo MY, Martelli AM, Murata RM, Rosalen PL, Bueno-Silva B, et al. Abilities of berberine and chemically modified berberines to interact with metformin and inhibit proliferation of pancreatic cancer cells. Adv Biol Regul. 2019; 73:100633. 10.1016/j.jbior.2019.04.003 31047842

